# 

**Published:** 1892-11

**Authors:** 


### LITERARY.

CONDENSED THOUGHTS ABOUT CHRISTIAN SCIENCE. By Dr. Wm. H. Holcome. The Purdy Publishing Co., 170 Madison street, Chicago. Price 25 cents.

The author is a "died in the wool" Christian Scientist. Fundamental propositions; "There is One God: even Jesus Christ," from Whom all life proceeds, hence all life is divine. All created things were good till man's appearance on Earth, endowed with free will, which rebelled against God, hence the origin of evil, not of God, but of man. The Spirit is the All in all. Matter has no real existence, it is merely a misconception, a delusion, the result of a perverted self will. Over all, the Spirit, if pure, has undisputed power. It can heal disease, because disease is an outgrowth of wrong thinking, of ignorance and misunderstanding of the Divine in us. There is but one substance, and that is, Spirit.

It is impossible to review satisfactorily a system which is grounded in error, whose premises are false or falsely conceived, whose basic statements are fallacies.

Surely *speculation* is the very opposite of *science*. To start out with a proposition which includes the Mosaic account of the Creation, and the theological deification of Jesus Christ, as basic truths upon which is reared all that follows, is the presentation of an unsolvable riddle, whose claim to any thing scientific is a glaring absurdity. No such miracle as Christ born of a Virgin, can be scientific, since its very essence is supernatural, or, rather, an affirmance of the supernatural, a quality which never was and never can be so long as God presides over the Universe and holds all created things, from the mightiest planet to the most infinitesimal atom, subject to His law, as immutable and unchangeable as God himself. But Christian Scientists will find comfort in this little book, for it is an affirmation of their worst absurdities.

The Dignity of Sex. By Henry S. Chase, M. D. Purdy Publishing Co., 168–170 Madison street, Chicago. 4to, 175 pages. Price 50 cents.

This is an admirable book, treating of the growth of the human race physically and mentally, from the embryo cell to perfect development, according to the unchangeable laws of being. It deals with the causes of physical and moral degeneration, and presents in painful contrast a vigorous manhood, the outgrowth of right living, right thinking and right doing, and the poor self-impoverished victims of unmanly abuses, to whom life itself is a continual reproach and a torture.

The work ought to be a text book in every family. The information it gives is too valuable to be withheld from the young of both sexes, as it usually is, out of a misconception of parental duty and obligation. By heeding its warnings and following its teachings the race would be greatly benefited.

Alaska; In Descriptive and Legendary Poems. By Professor Bushrod W. James, M. D., Porter & Couts, Publishers. 4to, 368 pp. Illustrated.

This is the most satisfactory book treating of our latest acquired territory, which has come under our notice. Although written in the language of the Muses, it deals with facts only. The extent, topography, and climatology of Alaska are truthfully portrayed in the metre of Hiawatha. Nor is this all. Its mountains, glaciers, great bays, rivers, animals, mineral deposits and vegetation, Indian tribes, their habits and legends, superstitions and local customs are truthfully portrayed in terms no less clear and concise for the embellishments of poetic phraseology. The work is amply illustrated, the entire volume is presented in a most skillful and artistic form, and cannot fail to meet with the favor of the reading public.

SECOND ANNUAL REPORT OF THE MIDWIFERY DISPENSARY, 314 Broome street, New York.

This is one of the worthiest charities of the western metropolis. Its beginning, under another name, was as early as 1799, and its then patrons include some of the most distinguished New Yorkers of that period. The work of the past year embraces 955 cases, successfully treated and disposed of.

CANCER, ITS CAUSES AND TREATMENT. By G. H. Stockham, M. D., Oakland, Cal.

This is a treatise of 45 pages. The author shows great familiarity with this troublesome and usually incurable disease.

QUEEN OF THE HOUSEHOLD MONTHLIES.—The October number of *Food* began its second volume. A prominent feature of this number is an illustrated story from the pen of the well known writer, J. H. Connelly, entitled the "Bulb of Grace." The entire contents are fully up to its well earned reputation as the leading household monthly.

